# Innovative problem solving in great apes: the role of visual feedback in the floating peanut task

**DOI:** 10.1007/s10071-019-01275-0

**Published:** 2019-07-05

**Authors:** Sonja J. Ebel, Martin Schmelz, Esther Herrmann, Josep Call

**Affiliations:** 1 Department of Developmental and Comparative Psychology, Max Planck Institute for Evolutionary Anthropology, Deutscher Platz 6, 04103 Leipzig, Germany; 2 School of Psychology and Neuroscience, University of St Andrews, St Mary’s Quad, South Street, St Andrews, Fife KY16 9JP Scotland, UK; 3 Department of Cognitive Biology, University of Vienna, Althanstrasse 14 (UZA1), 1090 Vienna, Austria

**Keywords:** Floating object task, Innovation, Primates, Social learning, Tool use

## Abstract

Nonhuman great apes show remarkable behavioural flexibility. Some individuals are even able to use water as a tool: They spit water into a vertical tube to make a peanut float upwards until it comes into reach (floating peanut task; FPT). In the current study, we used the FPT to investigate how visual feedback, an end-state demonstration and a social demonstration affect task performance in nonhuman great apes in three experiments. Our results indicate that apes who had acquired the solution with a clear tube maintained it with an opaque one. However, apes starting with an opaque tube failed to solve the task. Additionally, facing the peanut floating on a water-filled tube (i.e., an end-state demonstration) promoted success independent on the availability of visual feedback. Moreover, experiencing how water was poured into the tube either by a human demonstrator or by a water tap that had been opened either by the ape or a human did not seem to be of further assistance. First, this study suggests that great apes require visual feedback for solving the FPT, which is no longer required after the initial acquisition. Second, some subjects benefit from encountering the end-state, a finding corroborating previous studies.

**Electronic supplementary material:**

The online version of this article (10.1007/s10071-019-01275-0) contains supplementary material, which is available to authorized users.

## Introduction

Humans use a variety of tools in their daily lives and may have evolved specific cognitive adaptations for tool use (Csibra and Gergely 2009; Hernik and Csibra 2009; Vaesen 2012). While humans have long been considered to be the only species that manufactures tools, it is now well known that some other species such as chimpanzees or New Caledonian crows do so as well (Beck 1980; Shumaker et al. 2011). Innovative problem solving seems to develop late during human ontogeny with children becoming proficient by the age of six-to-eight years (Beck et al. 2011, 2012, 2016; Chappell et al. 2013; Cutting et al. 2011; Hanus et al. 2011). The term “innovation” is used with many meanings; one is invention and adoption by group members (Hochberg et al. 2017; Reader and Laland 2003), another one is innovation as producing a novel solution to a problem (Beck et al. 2011, 2012, 2014, 2016; Chappell et al. 2013; Cutting et al. 2011, 2014; Griffin and Guez 2014; Laumer et al. 2017; Nielsen 2013; Nielsen et al. 2014). The latter meaning is the one that we use in this paper.

More precisely, innovation problems are usually tasks in which the structure of the experimental setup does not provide information about the precise actions required to reach the goal state (i.e., ill-structured problems; Cutting et al. 2011; Jonassen 1997). These tasks demand a creative approach to finding a solution, which may involve the use of novel tools (Beck et al. 2011, 2012, 2014, 2016; Chappell et al. 2013; Cutting et al. 2011, 2014; Griffin and Guez 2014; Laumer et al. 2017; Nielsen 2013; Nielsen et al. 2014). However, relatively little is known about the evolutionary roots of innovative problem solving.

One task that has been used to study human children as well as our closest living relatives, the nonhuman great apes, is the floating peanut task (FPT; Hanus et al. 2011; Mendes et al.^2007^; Tennie et al. 2010). In the FPT, individuals have to pour water into a vertical clear tube which contains a peanut (or any other buoyant object) that floats upwards until it comes into reach. Recent studies have shown that human children performed decently by six-to-eight years of age (about 40–60% success) which is comparable to other innovation tasks; their performance increased with age when the tube already contained some water (Beck et al. 2011, 2014, 2016; Chappell et al. 2013; Cutting et al. 2011, 2014; Hanus et al. 2011). Moreover, when children were given a social demonstration, they copied the precise actions of the experimenter to fill the tube with water, involving an intermediate step that was causally irrelevant (i.e., filling water from a bottle into a cup first; Nielsen 2013). When great apes where tested in the same task, they generally used a different technique from children who used a bottle for transferring the water by pouring water from their mouths (Hanus et al. 2011; Mendes et al. 2007; Tennie et al. 2010).

Recent studies have shown that Sumatran orang-utans (*Pongo abelii*) and chimpanzees (*Pan troglodytes*) can solve the FPT (Hanus et al. 2011; Mendes et al. 2007), whereas none of the Bornean orang-utans (*Pongo pygmaeus*), gorillas (*Gorilla gorilla*) and brown capuchin monkeys (*Cebus apella*) tested did (Hanus et al. 2011; Renner et al. 2017). All Sumatran orang-utans (*N* = 5) solved the task spontaneously (and about 20% of the Bornean orang-utans showed unsuccessful spitting behaviour) and so did 21% of the chimpanzees (unsuccessful spitting behaviour: 17%; Hanus et al. 2011; Mendes et al. 2007). Interestingly, none of the chimpanzees from another population were successful at first with their familiar water dispenser, but when they were presented with a novel water dispenser, 11% solved the task (and 26% showed unsuccessful spitting behaviour; Hanus et al. 2011). The authors interpreted this evidence as a functional fixedness effect, that is, apes were fixated on the familiar function of the water dispenser which hindered them using it in a novel functional context (Duncker 1945; Hanus et al. 2011). Bonobos (*Pan paniscus*) have not been tested with this paradigm so far.

It is still unclear, however, whether successful subjects really anticipated the effect that spitting water into the tube would have on the peanut’s position or if they had added water to the tube for some other reason (e.g., to make contact with the peanut) and upon seeing its positive effects repeated the action until they managed to extract the peanut from the tube. Mendes et al. (2007) suggested using an opaque tube in the FPT to address this question. This would show if apes solving the task are aware of the process that would be occurring inside the opaque apparatus before they get feedback for their actions. The FPT requires repeated responses (i.e., minimized chance of discoveries by trial and error), but its complexity (or opacity) is not beyond apes’ capabilities so that it makes the task adequate to test apes’ anticipatory skills. This is an important consideration because if subjects were to succeed in such a task, it would indicate that visual feedback is not essential and would suggest that individuals can anticipate the effect that pouring water has on the position (and therefore accessibility) of the peanut.

Visual feedback can play an important role in problem solving (e.g., Köhler 1925; Taylor et al. 2010; Völter and Call 2012) and refers to any visual stimuli that serve as positive or negative feedback for an individual’s actions. This feedback helps to assess if the actions are likely to obtain the desired goal, e.g., when a chimpanzee is raking in a food reward with a stick, the chimpanzee can assess her/his progress by observing the food coming closer. Visual feedback generated by an individual’s own actions can facilitate or impede the appearance of an efficient solution to a problem. For instance, pushing an object away from the subject to overcome a barrier that is preventing its direct retrieval is difficult because subjects cannot resist bringing the object closer and consequently, after pushing it away, they repeatedly bring it back to the starting position (e.g., Guillaume and Meyerson 1930; Köhler 1925). The timing of feedback in relation to the solution, and not just its nature, is also important. For example, Taylor et al. (2010) presented New Caledonian crows with a vertical string pulling task in which they could pull up a string to which a piece of food was attached. All crows succeeded when they had full visual access to the string. Thereafter, the crows also solved a visually restricted version of the task in which a platform limited visual access. However, blocking visual feedback before first acquisition substantially hindered the solution and only one crow succeeded spontaneously (Taylor et al. 2010). Völter and Call (2012) presented nonhuman great apes with an analogous task in which apes could crank up a piece of food that was attached to a string inside an either clear or opaque apparatus (Völter and Call 2012). Some subjects spontaneously solved the task with the clear version, but all subjects failed with the opaque one. However, after apes had acquired the solution with the clear apparatus, they transferred it to the opaque one (Völter and Call 2012). Both studies suggest that while feedback was required for acquisition of the solution, it was no longer needed to maintain performance. An important question is if the impact of visual feedback would also be modulated by task difficulty. When Völter and Call (2012) presented apes with two less complex problems (i.e., pushing out a food item from a horizontal tube or removing sticks from a tower to release a food reward), the visual feedback was not necessary to succeed. However, apes were faster when visual feedback was available than when it was not, suggesting that visual feedback supports more efficient problem solving (Völter and Call 2012). These results highlight that the effect of visual feedback is modulated by task complexity and its timing (i.e., before or after the solution). Yet, the crank task might not be the best problem to investigate insightful anticipation since first, it employs a mechanism which is artificial and not easy to grasp for the apes and secondly, the task is still solvable by persistent manipulation of the apparatus. Thus, it would be interesting to further investigate the role of visual feedback in the FPT which meets both criteria (i.e., intuitive and not solvable with persistence alone).

Another study with apes assessed the effect of additional information in the FPT. Tennie et al. (2010) provided chimpanzees either with a full demonstration of the solution by a conspecific or by a human. They found no evidence that observing another ape solving the task was more useful than just observing the changes that occurred to the peanut’s location when a human experimenter poured water into the tube from a bottle (ape: 38% vs. bottle: 21%). Tennie et al. (2010) concluded that emulation learning explains the finding, that is, chimpanzees reproduced the end-state using their own actions, since there was no difference between a demonstration showing the precise actions required and a bottle demonstration (see also Call et al. 2005). Interestingly, chimpanzees also did not benefit more from encountering a tube that already contained some water (i.e., a partial solution) compared to a dry tube (Hanus et al. 2011).

In the current study we presented naïve chimpanzees with an opaque version of the FPT that prevented them from receiving visual feedback about the effect that spitting water into the tube had on the peanut’s position (Experiment 1). After a baseline with a dry tube, chimpanzees received some hints about how to solve the task: In the end-state condition, they encountered a water-filled tube with the peanut floating atop. In the human demonstration condition, they watched how the experimenter poured water into the tube until the peanut emerged at the opening. As the classical FPT is typically solved by a minority of apes only, we anticipated that depriving subjects of any visual feedback would make the task extremely difficult. Consequently, we complemented the opaque FPT experiment with two other experiments. We investigated whether bonobos and chimpanzees from another population would benefit from other kinds of feedback (Experiment 2). More specifically, we first established a baseline with the clear dry tube followed by three conditions that provided apes with additional information about task affordances (end-state, water tap turned on by the ape, water tap turned on by the experimenter). We further presented apes that had previously solved the clear tube with an opaque tube to find out whether disrupting visual feedback after acquisition affected their performance (Experiment 3). Therefore, we presented subjects with a clear dry tube first and then, with a dry opaque one. Furthermore, we investigated whether successful subjects confronted with an ineffective opaque tube differentially perseverated in pouring water depending on whether the cause of failure was visible or not. In the visual cause condition, subjects could see that the water escaped through a hole at the front of the tube while in the no visual cause condition, they did not receive such feedback as the water escaped at the back of the tube.

## Experiment 1

### Methods

#### Subjects

Twenty-four chimpanzees living at Sweetwaters Chimpanzee Sanctuary (Ol Pejeta Conservancy, Laikipia, Kenya) participated in the study. Among these were 14 females and ten males ranging from eight to 28 years (Table S1). The majority of chimpanzees was orphans, were born in the wild and came to the sanctuary after being confiscated from the illegal bushmeat and pet trade. They were all raised by humans in a highly comparable way, living together with peers after arriving at the sanctuary. Chimpanzees lived in two social groups with access to extensive outdoor enclosures and indoor sleeping rooms. They spent the day in the large outdoor enclosures and the night in the indoor sleeping rooms. The outside enclosures comprised bushland, trees and open areas. Since apes could exhibit many of their natural behaviours in this landscape (e.g., climbing or travelling long distances) and were part of a large social group, no special enrichment devices were needed to keep them busy throughout the day. The indoor sleeping rooms offered multiple platforms and materials for nest building. Chimpanzees were regularly fed throughout the study period and their diet comprised mainly local vegetables and fruits. All apes remained in their original housing locations at the conclusion of the study. Tests were conducted in the indoor sleeping rooms on a voluntary basis.

#### Materials

**Fig. 1:**
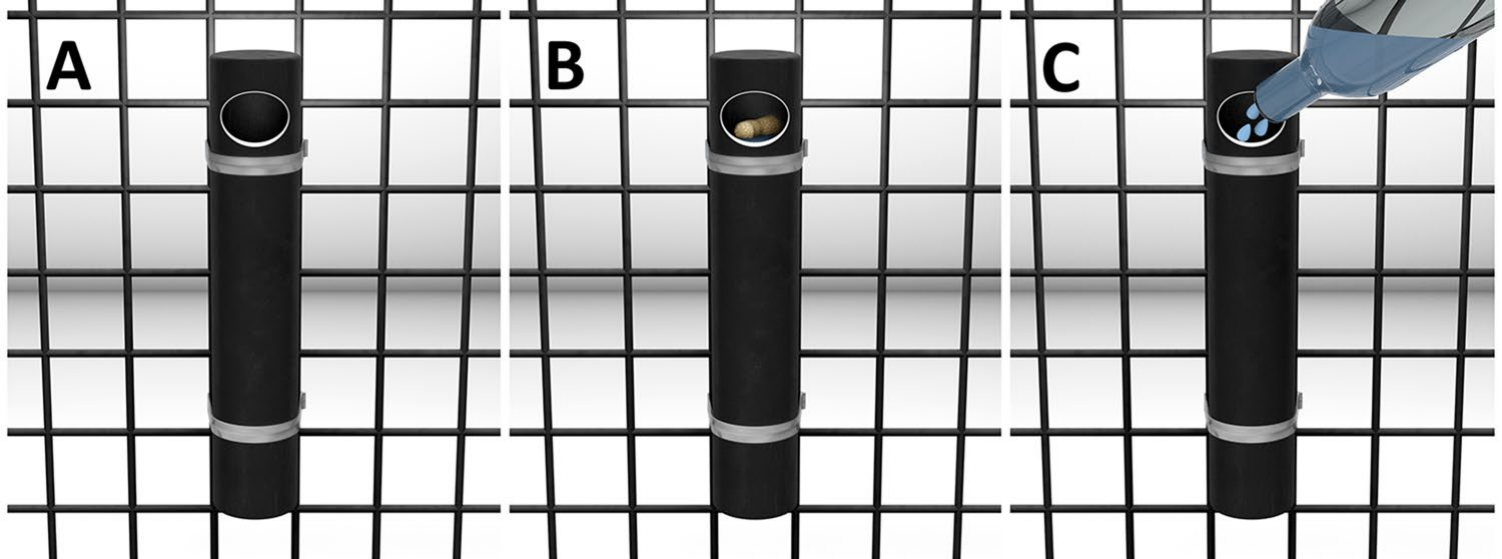
The three conditions of Experiment 1: baseline condition (**a**), end-state condition (**b**) and human demonstration condition (C)

We used an opaque Plexiglas tube (26 cm × 5 cm; outer diameter) that was closed at both ends. A hole (about 3 cm x 3.5 cm) was drilled on its upper front (Fig. 1). The size of the tube and the position of the hole were such that they blocked visual access to a peanut located at the bottom of the tube. In fact, the peanut became visible only as it neared the hole. We attached a water dispenser to a grey PVC plate about a metre away from the tube.

#### Procedure

Each chimpanzee received a maximum of six sessions with one session per day. After a chimpanzee had solved the task once, the chimpanzee did not receive any further sessions. First, the chimpanzee received two sessions with the baseline condition (Fig. 1a) followed by two sessions with the end-state (Fig. 1b) and two with the human demonstration condition (Fig. 1c). Since the study was part of a larger study which required the same order for all individuals, we did not counterbalance the order of conditions across individuals. Besides, we did not expect a massive improvement in the human demonstration condition as a previous study suggested that chimpanzees mainly benefit from encountering the end-state (Tennie et al. 2010). We conducted sessions on consecutive days (in exceptional cases some apes would not enter the test room and were then tested upon availability). We employed a human demonstration using a bottle (i.e., showing how to reach the outcome by a slightly different method) instead of one performed by a conspecific (i.e., showing the exact actions needed) because a recent study had shown that there was no difference between these two types of demonstrations (Tennie et al. 2010).

In the baseline condition, the chimpanzee watched from an adjacent room as the experimenter dropped a peanut into the tube. After entering, the chimpanzee had ten minutes to retrieve the peanut. In the end-state condition, the chimpanzee encountered the water-filled tube with the peanut floating atop which allowed them to retrieve the peanut from the water. After the chimpanzee had taken the peanut from the tube, the door to the adjacent room was opened and the subject left the room so that the experimenter could prepare the test trial. In the human demonstration condition, the chimpanzee witnessed from an adjacent room how the experimenter filled a bottle (capacity: 500 ml) with some water from the water dispenser and poured it into the tube. The experimenter repeated these actions three times until the tube was filled and the peanut came into reach (duration: about 60–90 s). Then, the chimpanzee entered the test room and could retrieve the peanut from the tube. After the chimpanzee had obtained the peanut in the end-state and the human demonstration conditions, the chimpanzee waited in an adjacent room until the experimenter had exchanged the wet tube for a dry one. Then, the sessions continued as in the baseline condition and the subject had 10 min to solve the task. The experimenter only performed actions (dropping the peanut into the tube in the baseline condition or pouring water into the tube in the human demonstration condition) when subjects were sitting at the mesh with their heads facing the tube. When subjects moved away, the caregivers called them and demonstrations continued as soon as they returned to their position. In this experiment as well as in the following two experiments, the apes were tested individually so that they could not observe the solution to the problem. Apes were separated from the group before entering the test room so that we were able to test specific individuals.

#### Coding and analyses

All sessions were videotaped. We coded success (i.e., retrieval of the peanut), latency to success, latency to first spit, number of spits and the mean inter-spit-interval using Solomon Coder (Péter 2011). In case subjects spat several times with one mouthful of water, this was still counted as one spit.

### Results

None of the chimpanzees acquired the solution or added water to the tube during the baseline sessions. After receiving an end-state demonstration, one chimpanzee solved the task without ever having seen someone adding water to the tube before (Jane, session 4). Thus, she solved the task without attaining visual feedback for her actions. Remarkably, she continuously added water to the tube without pausing once. Two additional females added water to the tube after an end-state demonstration, but not enough to obtain the peanut (Cheetah, session 3 + 4; Julia, session 4). After her first and second spit in session 3, Cheetah found a vegetable stalk and inserted it into the tube repeatedly for about a minute. Thereafter, she quit the task. It is possible that the stick-like object distracted her from adding more water to the tube. Interestingly, all spitting behaviour except for one event (Cheetah, session 3) occurred very late in the session, after more than 8 minutes (see Supplemental Material for more details). None of the chimpanzees added water to the tube in the human demonstration condition. The three chimpanzees who added water to the tube were 11, 15 and 28 years old (mean age of sample: 18 years).

### Discussion

One chimpanzee solved the FPT when visual feedback was blocked after receiving information about the end-state of the task, i.e., encountering a water-filled tube with a peanut floating atop. Thus, at least one individual solved the task without receiving any immediate visual feedback for her spitting actions and without ever having seen water being poured into the tube before. This perseveration constitutes the first evidence, albeit weak, that an individual may have anticipated the consequences of her actions in the FPT. No other subjects showed this behaviour and the two chimpanzees who added water to the tube once or twice quit, perhaps because they obtained no feedback. Moreover, none of the chimpanzees acquired the solution during the baseline, suggesting that apes require visual feedback to solve the FPT spontaneously since at least some apes solved the task spontaneously in previous studies (Hanus et al. 2011; Mendes et al. 2007). Besides, chimpanzees were not further benefitting from a human demonstration, that is, none of the subjects added water to the tube in this condition. Due to a floor effect we could not run statistical analyses so that generalizations from these results are limited. However, the findings are consistent with the idea that end-state information facilitated spitting behaviour in some individuals in the FPT when visual feedback was blocked. Although emulation and imitation learning were not directly compared in the current study, findings corroborate the idea that emulation learning was enough to explain chimpanzees’ success in a previous study (Tennie et al. 2010: see also Call et al. 2005).

One possibility why the human demonstration did not increase success rates in chimpanzees is that subjects received no bottle, thus preventing them to imitate the precise actions that the demonstrator performed. Although we do not know what chimpanzees would do when given the bottle, we consider it unlikely that success rates would increase for three reasons. First, observing a demonstration by another chimpanzee using her mouth in the FPT was as effective as observing a human employing the bottle in a previous study (Tennie et al. 2010). Second, using a bottle to transport water (i.e., using a tool to transport a tool) is likely to be more difficult than using one’s mouth (i.e., transport the tool itself). Chimpanzees often carry objects and food items in their mouths, but rarely use containers to transport objects. Third, from the point of view of motor skills, filling the bottle with water and emptying its contents into the tube might be more challenging than spitting it from the mouth.

Furthermore, conditions differed in regard to their reinforcement structure. While subjects’ actions were reinforced during the end-state and the human demonstration conditions when they retrieved the peanut from the tube, they were not reinforced during the baseline condition. Such reinforcement might have led to a higher motivation to engage with the task thus providing a better explanation than emulation learning for the spitting behaviour of the three individuals. However, first, a recent study showed a positive effect of social demonstrations in the FPT, even though apes were not reinforced directly during these demonstrations since a dominant conspecific was in the same room with the subject and always took and ate the peanut (Tennie et al. 2010). Second, chimpanzees readily manipulated the tube and the water dispenser during baseline sessions, something that indicates that they were motivated to engage with the task. Yet, it is still possible that observing a conspecific accessing and eating a peanut might have a comparable reinforcing effect as eating the peanut oneself, an issue that requires further investigation. Note, however, that reinforcement may also play a role in emulation learning in natural settings where food leftovers, which can be considered in some cases “end-states”, may act as reinforcers. Future studies could directly compare the relationship between end-state and social demonstrations on the one hand and the reinforcement that subjects experience themselves or observe in others on the other hand.

One reason why the pouring demonstrations in the current and previous studies might not have been more effective is that the change in the position of the peanut was not caused by the subjects’ own actions. In other words, if the subject had caused the change and not been a mere observer of both the cause and the effect, this would have been more effective in producing a solution. Therefore, in the next experiment we devised a task in which the subject caused the change during the demonstration phase, but using different means of what the subject would be required to do during the test phase (i.e., pour water from her/his mouth into the tube). More specifically, we presented chimpanzees and bonobos with the clear version of the FPT followed by three conditions: an end-state demonstration condition, a condition in which they themselves activated a tap that filled the tube with water and brought the peanut within reach and a condition in which the experimenter activated the tap.

## Experiment 2

### Methods

#### Subjects

Six bonobos and 18 chimpanzees participated in the study (Table S2). All apes were housed at the Wolfgang Köhler Primate Research Centre (WKPRC) at Leipzig Zoo (Leipzig, Germany). Among these were 19 females and five males ranging from six to 48 years of age. Eleven were nursery-reared, ten were mother-reared and the rearing history of three was unknown. While all six bonobos and five chimpanzees were naïve to the FPT, 13 chimpanzees had been presented with this task in previous studies, but had failed to solve it (Hanus et al. 2011; Tennie et al. 2010; Table S2). All subjects lived in social groups of various sizes with access to indoor and outdoor enclosures that comprised various enrichment devices such as trees and ropes to climb on, shaking boxes and poking bins. Additionally, apes were fed enrichment materials in the afternoon (e.g., wrapped up food in paper or jute). They stayed in their indoor sleeping rooms during the night and spent their day in the indoor or outdoor enclosures depending on the weather. Apes were fed with vegetables, fruits and sometimes eggs or meat; thus, they were neither food, nor water deprived at any time throughout the study. Participation in the study was voluntary and any food gained was additional to their daily diet. Testing took place individually in the apes’ indoor sleeping rooms and all apes remained in their original housing locations at the conclusion of the study. We stopped testing (and excluded from the analyses) two chimpanzees (Annett, Swela) because one refused to eat wet peanuts and one did not pull the string in the additional information phase (*N*_final_= 22).

#### Materials

**Fig. 2:**
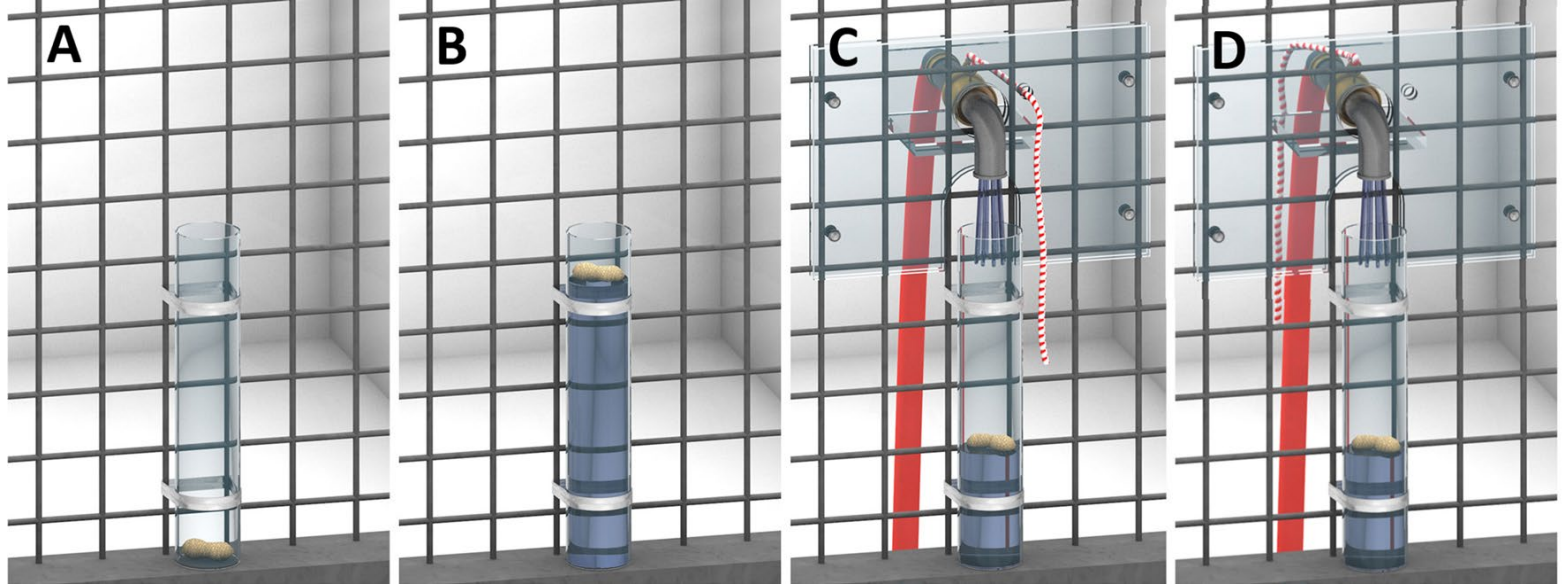
The four conditions of Experiment 2: baseline condition (**a**), end-state condition (**b**), water tap by ape condition (**c**) and water tap by human condition (**d**)

We used a dry clear Plexiglas tube (26 cm × 5 cm; outer diameter) that was closed at the bottom with a peanut inside (Fig. 2). For the bonobos, we could not use peanuts due to a peanut allergy of one individual. We assessed bonobos’ preference for dried pieces of apple and banana and used their preferred food item for the test. A black steel container (50 cm × 20 cm × 30 cm) filled with water was attached to the mesh (same as in Allritz et al. 2013). We used an open water source instead of a water dispenser. Although our choice made this experiment not comparable to Experiment 1, our goal here was not to compare experiments but to potentially increase the likelihood of success by increasing the salience of the water. The distance of the water to the tube varied across the ape groups due to the conditions of the respective sleeping rooms (bonobo, chimpanzee group 1: about 2.25 m, chimpanzee group 2: about 1.40 m). In the “water tap by ape” and “water tap by human” condition, a water tap was attached above the tube (see Fig. 2c, d; Fig. S1). The tap could be opened by pulling a string so that water would flow into the tube. By pulling the string, a metal ring at the tap was moved towards the ape which produced a banging sound when the movement stopped. The string-pulling was actually non-functional and the water was turned on by the experimenter operating a valve out of sight.

#### Procedure

Apes received a maximum of eight sessions with one session per day. When apes solved the task once, they were not given further sessions. First, they received two sessions with the baseline condition followed by six sessions in which they received additional information (end-state, water tap by ape and water tap by human), counterbalanced for order across individuals. Each of the additional information conditions was given on two successive sessions. We administered all sessions on separate days either on consecutive days or a few days apart depending on other tests carried out at WKPRC. In the baseline condition, apes were presented with a dry tube containing a peanut (or a piece of dried apple or banana in case of the bonobos) for 10 min. In the end-state condition, apes encountered a tube filled with water and the peanut floating atop (Fig. 2b). In the water tap by ape condition, apes faced a tube with a peanut located inside. They could pull a string which moved a metal ring to “turn on” the water, while the experimenter actually operated a valve (Fig. 2c). When the tube was filled with water, they could retrieve the peanut from the tube. In the water tap by human condition, the water was turned on by the experimenter by moving the metal ring herself in view of the ape (Fig. 2d). Each session in the additional information phase consisted of three demonstrations, followed by the original test with a dry tube. Between the demonstrations the experimenter emptied the tube and placed a new peanut inside. After the demonstrations the water tap was removed and the wet tube was exchanged for a dry one. While the setup was prepared, subjects waited in an adjacent room. Subjects were tested individually. However, in exceptional cases another individual was inside the test room area to ensure that apes felt secure (e.g., sometimes a juvenile was tested with her mother inside), but there were one or more rooms in between the two individuals and/or occluders were put up to ensure that apes could not watch the solution or even the subject getting water from the water source (or the demonstrations).

#### Coding and analyses

All sessions were videotaped. We coded success (i.e., retrieval of the peanut), latency to success, latency to first spit, number of spits and mean inter-spit-interval using Solomon Coder (Péter 2011). As before, in case subjects spat several times with one mouthful of water this was still counted as one spit. A second coder coded all videos with spitting behaviour from Experiments 2 and 3 and reliability was excellent (Pearson’s correlation coefficient: latency to success, *r* = 0.996, *df* = 20, *p* < 0.001; latency to first spit, *r* = 0.993, *df* = 50, *p* < 0.001; number of spits, *r* = 0.995, df = 50, *p* < 0.001).

### Results

Two chimpanzees solved the FPT in the baseline sessions (Kofi, session 1; Sandra, session 2). Three additional chimpanzees acquired the solution in the additional information phase, with two of them succeeding in the first session. More specifically, one subject solved the task after an end-state demonstration (Lobo, session 3) and one after activating the water tap herself (Alexandra, session 3). Moreover, one chimpanzee solved the task after an end-state demonstration (Kara, session 5), after she had already passed two unsuccessful sessions with the water-tap by human condition. One additional chimpanzee and one bonobo added water to the tube, but not enough to obtain the peanut (Riet, baseline; Fimi, baseline and water tap by ape; see Table 1 and Table S3). One subject employed two additional techniques to add water to the tube next to spitting: Kofi peed into the tube and used his hand once to transport the water. Interestingly, all successful apes (mean age: 13 years) were younger than the mean age of our sample (23 years).

### Discussion

Five chimpanzees solved the FPT by repeatedly spitting water into the clear tube until they could reach the peanut. Two of them did so spontaneously during the baseline while the other three solved the task after receiving additional information about the solution that always comprised the end-state (i.e., the peanut floating on a water-filled tube). Another chimpanzee and one bonobo added water to the tube, but not enough to extract the peanut. This finding corroborates the results from Experiment 1 with an opaque tube and the ones by Tennie et al. (2010) with a clear tube which showed that chimpanzees benefited from encountering the end-state in the FPT, although the floor effect in Experiment 2 again limits the generalizations that can be drawn from these findings alone (but see also Vale et al. 2017 for the positive effect of model demonstrations on the occurrence of solutions). We found no evidence of a difference with regard to success between conditions in which the ape or the human controlled the water tap to fill the tube, but the low success rate makes the interpretation of this result difficult. Future research could investigate whether the end-state produced by the individual’s own action versus the action of someone else affect the likelihood of learning by emulation.

While our results showed very low success rates in the FPT with a clear tube, a word of caution in interpreting these results is necessary. Some of our subjects had participated in previous studies using the FPT, but had failed the task while previously successful individuals who might have had a greater potential to solve the task were not included (Table S2; Hanus et al. 2011; Tennie et al. 2010). Furthermore, it is difficult to compare the baseline to the conditions in the additional information phase given that their order of presentation was not counterbalanced across subjects. However, this was not the goal here. A previous study had already established that additional baseline sessions did not improve performance (Hanus et al. 2011). More specifically, solutions typically occurred in the first or second baseline sessions or not at all (Hanus et al. 2011). The fact that three additional individuals apparently benefited from end-state conditions is, therefore, entirely consistent with previous studies.

To our knowledge we tested bonobos for the first time with the FPT. Although none of the six individuals solved the task, one subject added water to the tube in two sessions, but not enough to obtain the dried piece of fruit. However, why no bonobo in comparison to chimpanzees solved the task remains an open question. Future studies could investigate factors like the difference in food reward, or differences in persistence and food motivation across species.

Our findings further support the idea that certain forms of visual feedback facilitated the solution in the FPT. As a next step, we assessed whether apes maintained the solution in the FPT with an opaque tube. In Experiment 1, one ape potentially anticipated the effect of the water on the peanut’s position, but all other subjects provided no evidence. Here we investigated whether apes would continue solving the task (by repeatedly pouring water in the tube) despite not being able to see the peanut moving upwards. This is equivalent to the manipulation by Taylor et al. (2010) in the string pulling task and Völter and Call (2012) in the crank task. To do so, we presented chimpanzees and orang-utans who had already acquired the solution in the FPT with a clear tube and the opaque tube that we used in Experiment 1. In a final manipulation we confronted successful apes with the opaque tube with a hole drilled near the bottom so that any water that was poured into the tube escaped via this hole, thus, preventing the peanut from moving upwards. In the “visual cause” condition, the water escaped from a hole at the front, thus providing information about the cause for the peanut’s lack of upward movement. In the “no visual cause” condition, the water escaped from a hole at the back of the tube, out of sight of the apes. We examined if apes would change their behaviour (i.e., stop adding water to the tube) because of the visual feedback that they received and that contained information about the tube’s malfunctioning.

## Experiment 3

### Methods

#### Subjects

Eight chimpanzees and five Sumatran orang-utans participated in the study (Table S4). They included nine females and four males ranging from nine to 25 years of age. Twelve of the 13 apes were mother-reared while the remaining one was nursery-reared. Apes were housed at the WKPRC (see Experiment 2 for housing conditions) except for two orang-utans who were housed at Zoo Dortmund (Dortmund, Germany). These two orang-utans had access to indoor and outdoor enclosures and lived in a social group. They participated voluntarily in the study by entering the sleeping room and engaging with the task. The orang-utans were regularly fed throughout the study period. They were neither water, nor food deprived so that the food they gained in this study was additional. All apes remained in their original housing locations at the conclusion of the study.

Subjects had previously solved the FPT with a clear tube except for one orang-utan (Tao) who as an infant had instead witnessed her mother solving the task (see Supplemental Online Material for more details). We also tested this subject to see if she remembered observing her mother solving the task (clear tube) and to increase our sample size (opaque tube). Subjects varied in the amount and the timing of their successful experience with the FPT. While the five chimpanzees from Experiment 2 had solved the FPT only once about 1 month before, the other subjects had solved the task several times, but several years ago (see Supplemental Online Material for more details on the individual testing backgrounds).

#### Materials

**Fig. 3:**
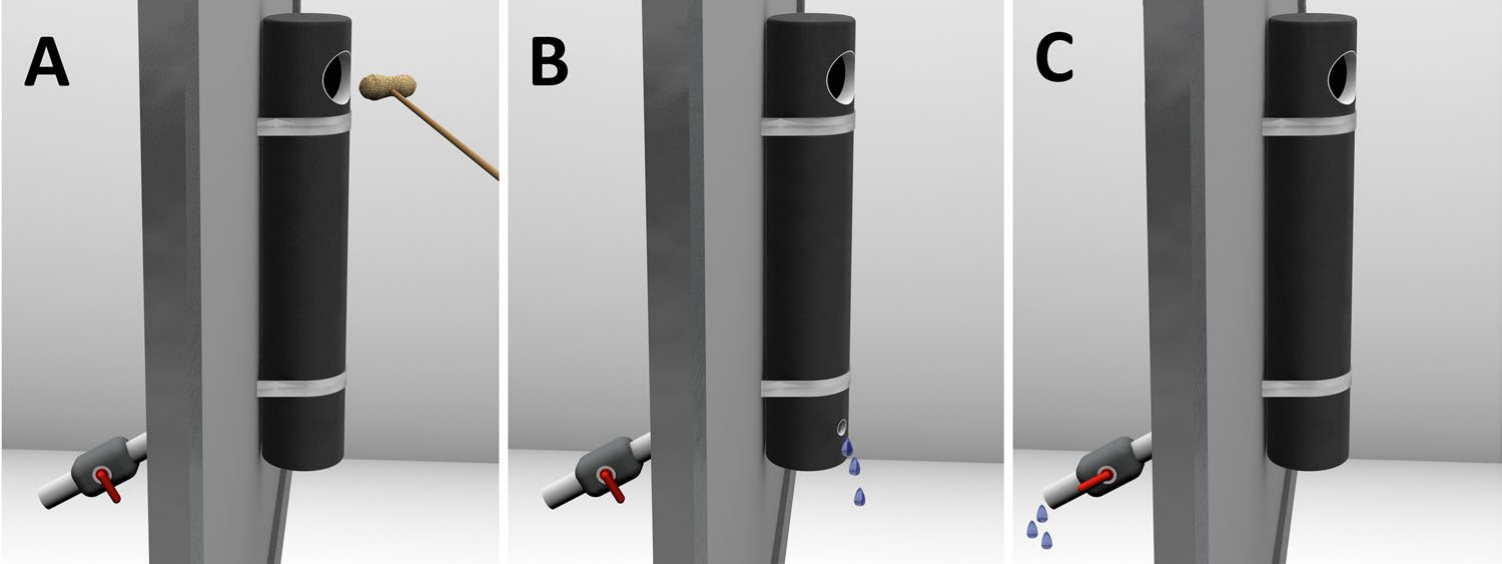
The three conditions of Experiment 3: opaque condition (**a**), visual cause condition (**b**) and no visual cause condition (**c**)

We used the same clear tube as in Experiment 2. Additionally, we used a modified version of the opaque tube from Experiment 1 that included the following changes (see Fig. 3): The tube was glued to a Plexiglas plate and a hose was attached to its back. The hose was connected to a valve that could be switched on and off. It was closed throughout the opaque condition to prevent the water from escaping the tube (Fig. 3a). The mesh surrounding the tube was covered so that apes could not see behind the tube. Apes were tested with the water source that was used when they first acquired the solution. Thus, we either used the black steel container from Experiment 2, a novel water dispenser or the familiar water dispenser. The distance of the water to the tube varied across the ape groups due to the different water sources and conditions of the sleeping rooms (chimpanzees: about 2.25 m or 1.40 m, orang-utans: about 2.25 m + 2 m in height). Due to experimenter error, we tested one chimpanzee (Frodo) with the familiar water dispenser although this individual had been tested with a novel one before (note that this chimpanzee still added water to the tube, yet, not enough to obtain the peanut).

#### Procedure

Apes received a maximum of four sessions with one session per day. We administered all sessions on separate days either on consecutive days or a few days apart dependent on other tests carried out at WKPRC. However, we always administered the clear condition and the opaque condition on consecutive days (i.e., about 24 h between success in the clear condition and presentation with the opaque condition). We, therefore, administered one additional session with the clear tube with one orang-utan (Padana) to ensure the same timing between conditions (the data of the first session is presented in the results section). Each session lasted a maximum of 10 min. First, we presented apes with the clear tube followed by the opaque tube, each for two sessions. As soon as they solved the task in a given condition, they did not get a second session with that same condition. Only apes who solved the clear tube received the opaque tube. One orang-utan (Toba) broke off the bottom of the clear tube in her first session and we repeated the session on the next day (see Supplemental Online Material for more details).

Three orangutans (Dokana, Padana, Raja) received two additional sessions with the opaque tube prior to the clear tube, resulting in a maximum of six sessions for these subjects (2 opaque, 2 clear, 2 opaque). We did so because these individuals had already been re-tested with the clear tube in a recent study in which they all solved the task (unpublished data). Thus, we confronted them directly with the opaque tube. Since none of them solved the opaque tube in the first two sessions, we decided to give them the same procedure as the other apes (i.e., giving them two more sessions with the clear tube and then, two with the opaque tube). We made this decision because the time frame for maintaining the solution from the clear to the opaque tube was about 24 h for most of the apes while it was much longer for these three individuals.

In the clear condition, upon entering the room the ape encountered the peanut located inside a dry and clear tube (Fig. 2a). In the opaque condition, the experimenter placed a peanut near the opening of the tube with the aid of a stick which was dropped when the subjects approached the apparatus (see Fig. 3a). In comparison to Experiment 1, a stick was used to avoid any physical contact with the ape since the ape was in the same room as the apparatus. Upon completion of the main phase of the experiment, apes who had been successful with the opaque tube received a follow-up test composed of two conditions presented in separate sessions with the order of presentation counterbalanced across individuals. In the “visual cause” condition, the opaque tube had a hole located at its lower front so that any water poured into the tube escaped through it (Fig. 3b). In the “no visual cause” condition, the hole was located at the back of the tube hidden from the subject’s view (Fig. 3c). Both conditions were impossible to solve. Sessions lasted 10 min each.

#### Coding and analyses

We videotaped all sessions and scored success (i.e., retrieval of the peanut), latency to success, latency to first spit, number of spits, latency to first spit and the mean inter-spit-interval using Solomon Coder (Péter 2011). As before, in case subjects spat several times with one mouthful of water this was still scored as one spit. We compared conditions for successful subjects performing Exact Wilcoxon signed rank tests in R (Hothorn and Hornik 2015; R Core Team 2013). We analysed the two species pooled together due to the small sample size and because there is no clear evidence of a species difference so far with the FPT (Hanus et al. 2011; Mendes et al. 2007).

### Results

#### Clear tube

Ten out of 13 apes solved the FPT with a clear tube in the re-test (77%; six chimpanzees and four orang-utans), i.e., they added enough water to obtain the peanut (two chimpanzees and one orang-utan were unsuccessful; see also Tables 1 and 2). More specifically, seven individuals solved the task in the first session and three in the second one. Two of these three individuals (two orang-utans: Toba, Tao) had been transferred to a new holding facility and it could be that they were not aware of the water dispenser in the test room during the first session. Additionally, one of these two orang-utans (Toba) broke off the bottom of the tube in the first session after she had repeatedly spat saliva into the dry tube. Both individuals were provided with a water bucket in the second session and then, solved the task (see Supplemental Online Material for more details).

**Table 1:** Results of Experiment 1–3: Success and spitting behaviour (i.e., successful and unsuccessful spitting behaviour summarized) in the FPT. Sample sizes differ between conditions because apes did not receive any further sessions after they had solved the task once

Experiment 1: opaque tube; naive chimpanzees^a^				
	Baseline	End-state	Human demo	
Success	0/24	1/24	0/23	
Spitting	0/24	3/24	0/23	

Experiment 2: clear tube; naive and previously unsuccessful chimpanzees and bonobos^b^				
	Baseline	End-state	Water tap by ape	Water tap by human
Success	2/24	2/21^c,d^	1/20^c^	0/20
Spitting	6/24	2/21	2/20	1/20

Experiment 3: clear and opaque tube; previously successful chimpanzees and orang-utans^a^				
	Clear	Opaque		
Success	10/13	7/10		
Spitting	12/13	9/10		

^a^Fixed order of the conditions

^b^Counterbalanced order of the demonstration conditions with two sessions of each condition given consecutively (baseline fixed)

^c^Successful in the first demonstration condition that the subject received

^d^Successful in the second demonstration condition that the subject received

**Table 2:** Results of Experiment 3 by species (only successful sessions included); median (range)

Species	Condition	Number of spits	Latency until success [sec.]	Latency until first spit [sec.]	Mean inter-spit-interval [sec.]
Chimp (*N* = 6)	Clear	6 (4–8)	125 (23–239)	22 (3–194)	12 (4–30)
Chimp (*N *= 5)	Opaque	4 (3–8)	219 (146–289)	54 (33–120)	28 (10–84)
Chimp (*N* = 5)	Front	24 (4–35)	NA	17 (4–130)	30 (17–80)
Chimp (*N* = 5)	Back	10 (7–18)	NA	27 (14–61)	38 (29–86)
Orang (*N* = 4)	Clear	3 (2–5)	134 (66–280)	74 (18–137)	28 (22–32)
Orang (*N* = 2)	Opaque	2 (2–2)	217 (121–313)	83 (35–130)	93 (29–156)
Orang (*N* = 2)	Front	5 (4–6)	NA	46 (25–67)	98 (60–136)
Orang (*N* = 2)	Back	3 (3–3)	NA	55 (41–70)	232 (230–234)

Three subjects remained unsuccessful in the re-test with the clear tube, yet, two of them added water to the tube. One orang-utan’s (Raja) failure was caused by wood wool that she stuffed into the tube. Although she subsequently filled it with water to the top, the peanut got stuck by the wood wool and, therefore, she failed to retrieve it. On the second session, she quit after three spits, thus, failing the task. One chimpanzee (Frodo) added five mouthful of water in his second session, but failed to obtain the peanut as it was not close enough to the opening yet, while another chimpanzee (Lome) did not add any water to the tube. The successful subjects were comparable in age with the unsuccessful ones (successful: mean = 15 years, range = 9–25; unsuccessful: mean = 14 years, range = 10–20), but the sample size was too small to draw any further conclusions.

#### Opaque tube

Seven out of ten apes solved the opaque tube (70%; five chimpanzees and two orang-utans), i.e., they added enough water to the tube to obtain the peanut without receiving visual feedback for their actions (one chimpanzee and two orang-utans were unsuccessful; see also Tables 1 and 2). Six of them did so in the first session and one in the second session. Moreover, one unsuccessful orang-utan (Dokana, first presentation of the opaque tube) and one unsuccessful chimpanzee (Sandra) added water to the opaque tube, while one orang-utan (Tao) did not add any water. The successful subjects were comparable in age with the unsuccessful ones (successful: mean = 13 years, range = 9–20; unsuccessful: mean = 18 years, range = 9–25), but the sample size was too small to draw any further conclusions. All three orang-utans (Dokana, Padana, Raja) who received two additional sessions with the opaque tube *before* they encountered the clear tube (and then the opaque tube again, see methods) added water to the opaque tube in the first two sessions, but none of them solved the task within these first two sessions.

There was no difference between the clear and the opaque conditions with regard to latency to success (Wilcoxon test: T = 22, *p* = 0.219, *N* = 7), latency to first spit (Wilcoxon test: *T* = 20, *p* = 0. 343, *N* = 7), mean inter-spit-interval (Wilcoxon test: *T* = 18, *p* = 0.156, *N* = 6, one tie) or number of spits (*N* = 5, two ties; no Wilcoxon test possible due to small sample size).

#### Follow-up test

There were significant differences in spitting frequency between the baseline and experimental conditions (Friedman test: *χ*^2^ = 9.0, *df* = 2, *p* = 0.011, *N* = 7). Subjects spat significantly less often in the baseline compared to the front and back conditions (Wilcoxon test: *T* = 21, *p* = 0.031, *N* = 6 in both cases), but there were no significant differences between the front and back conditions (Wilcoxon test: *T* = 22, *p* = 0.234, *N* = 7). However, subjects spat more often in the first compared to the second experimental condition that they received (Wilcoxon test: *T* = 26.5, *p* = 0.047, *N* = 7). There were also significant differences between conditions with regard to the mean spitting frequency (Friedman test: *χ*^2^ = 6.0, *df* = 2, *p* = 0.050, *N* = 7). However, pairwise comparisons failed to confirm the differences between conditions (Wilcoxon tests: *T* < 25, *p* = 0.109, *N* = 7 in all cases). Similarly, there was no significant difference in the latency to spit between the first and the second conditions that subjects received (Wilcoxon test: *T* = 21, *p* = 0.300, *N* = 7). The descriptive statistics for each species are reported in Table 2.

### Discussion

Chimpanzees and orang-utans who acquired the solution of the FPT one month to nine years before, solved the task again upon presentation. Moreover, most of the successful subjects transferred the solution to an opaque tube that deprived them of visual feedback, i.e., they could not perceive the effect that their spitting actions had on the peanut’s position. These results suggest that apes were able to reproduce the solution to a problem after a long period of time since the first solution and that the ability to solve this task was independent of visual feedback after first acquisition.

Apes continued to solve the task after they were deprived of visual feedback despite having to perform repeated actions over the course of about two-and-a-half minutes (from first spit to the retrieval of the peanut) without being able to assess if their manipulation was successful. While seven apes (70%) showed this high level of persistence, three apes (30%) failed to transfer the solution to the opaque tube. Since two of them still added water to the tube, visual feedback might have been essential for them to solve the task. These results, taken together with those of Experiment 1, are consistent with the findings of two recent studies, one with a cranking task in great apes and one with a vertical string pulling task in New Caledonian crows (Taylor et al. 2010; Völter and Call 2012; see also Vale et al. 2016). In these studies, some individuals acquired the solution when visual feedback was available and also transferred it to an apparatus that restricted or completely blocked visual feedback. However, none of them (except for one crow) acquired the solution when visual feedback was restricted or blocked, like in Experiment 1 (Taylor et al. 2010; Völter and Call 2012). One major difference between these studies and the current one is that the FPT is not solvable by persevering with a manipulation at the apparatus, but requires relating a seemingly non-related object (i.e., the water dispenser or the water) to the task. Although the mechanism was more natural and did not comprise a human-made mechanism such as cranking, apes did not show evidence that they anticipated the outcome of their actions in the FPT.

Apes solved the FPT although they had not faced the task for a period that ranged from one month up to nine years. Although this may be an indication of good memory performance, it may also be a sign of problem-solving consistency. That is, those individuals who solved the task originally, also solved it (independently) a few years later and without necessarily recalling that solution. Without comparing the initial latencies to solve the task with the latencies in the current study it is unclear whether their success represents a case of good memory or re-innovation. Although this would have been a desirable comparison, we were unable to carry it out because first, only five subjects solved the task in Experiment 2 and second, overall few subjects have solved the task spontaneously in Experiment 2 or in previous studies which makes it difficult to assess latencies (but see Vale et al. 2016).

When subjects faced failure in the follow-up test, they perseverated in adding water to the tube although their attempts substantially decreased in the second session. Indeed, the order of presentation of the conditions rather than the conditions themselves (i.e., seeing the cause of failure or not) seemed to be the factor that best explained subjects’ reduction in spitting frequency. In contrast, mean spitting frequency did not differ between conditions. These findings suggest that apes did not take into account visual feedback about the cause of their failure because they did not decrease their spitting behaviour when they could see the water flowing out of the tube. However, a larger sample would be needed to analyse this in greater detail as few generalizations can be made from such a small sample.

## General Discussion

We found that visual feedback can play a pivotal role in the initial acquisition of the solution to an innovation problem in great apes, but decreases its importance thereafter. While apes were able to solve the task again after a period of time (ranging from one month to nine years) with the clear tube and then transferred the solution to an opaque tube that completely blocked visual feedback (Experiment 3), they failed to solve the task when visual feedback was absent when they first encountered the task (Experiment 1). Additionally, the type of feedback about their failure (i.e., a seen or unseen cause) did not alter their spitting behaviour, i.e., when the water escaped through a hole at the front or back of the tube (Experiment 3). Intriguingly, observing the solution led to success in some individuals who had not been successful before: some apes who experienced a water-filled (clear or opaque) tube solved the task subsequently while experiencing how the water was added to the tube by a human demonstrator or a water tap did not seem to be of further assistance (Experiments 1, 2). However, generalizations about social learning in apes are limited from these specific findings, since no statistical analyses were possible due to few subjects solving the task overall.

One individual solved the opaque tube after experiencing an end-state demonstration, that is, she solved the task without experiencing the effect that adding water to the tube had on the peanut’s position. This provides some (albeit weak) evidence that one subject may have anticipated the outcome of her actions in the FPT. However, the FPT may have been too difficult for apes to show their anticipatory abilities (see also Redshaw and Suddendorf 2016; Völter and Call 2014). Even in its easier version (clear tube), only a minority of apes solved the task (Hanus et al. 2011; Tennie et al. 2010). Furthermore, the task required the ability to delay gratification as well as the necessary motivation to continue spitting despite not obtaining anything. Although apes generally perform well in delay of gratification tasks and can wait for 60–180 s to get a higher valued reward (Beran 2002; Rosati et al. 2007), not seeing any change in the peanut’s position may have discouraged them. Recall that apes needed on average about 150 s from their first spit to retrieve the peanut from the opaque tube in Experiment 3. Therefore, we must interpret our results with caution. Apes may be able to anticipate the outcome of their actions with easier tasks since studies have shown that they possess some future planning abilities (Janmaat et al. 2014; Mulcahy and Call 2006; Osvath and Osvath 2008; van Schaik et al. 2013; Völter and Call 2014).

Our findings are consistent with previous studies showing that great apes and New Caledonian crows were dependent on visual feedback for acquisition, but not for maintenance of the solution in a string pulling task (Taylor et al. 2010; Völter and Call 2012). Acquisition of the solution in the FPT could be based on different underlying processes. First, apes might have solved the FPT by anticipating the outcome of their actions and some form of causal understanding (see also Köhler 1925; Völter and Call 2014; Völter et al. 2016). We found little evidence for this (see Redshaw and Suddendorf 2016). Second, apes may have added water to the tube to move the peanut intentionally (i.e., acted on a creative idea; see Bateson 2014) and then, were differentially reinforced by visual feedback (i.e., the peanut nearing the tube’s opening). Third, apes may have solved the task by trial-and-error learning and differential visual reinforcement. In this case, they would have added water to the tube by chance resulting in differential reinforcement. We consider the second alternative somewhat more likely than the third one because spitting into the tube is a novel and unusual response (recall that overall only few apes spat into the tube, see also Hanus et al. 2011; Tennie et al. 2010). Moreover, apes sometimes solve tasks apparently with little visual feedback about task affordances and/or without any evidence of learning by inferring the task’s causal structure (Boesch 2013; Hanus and Call 2008, 2011; Völter et al. 2016).

Most apes solved the task again after months and even years after the original solution. This finding can again be interpreted in different ways. First, this may be an indicator of intra-individual consistency in problem solving and apes may have re-innovated when presented with the task for a second time. However, it is an open question which characteristics would classify an innovator in the FPT. A recent study with human children showed that success in another innovation problem (the hook task) was not predicted by divergent thinking or executive functions such as inhibition, working memory, or attentional flexibility (Beck et al. 2016). Yet, this study found that a measurement that is potentially associated with general intelligence predicted success. Second, apes’ success may be an indicator of excellent long-term memory. They potentially remembered the solution, e.g., via cued recall when facing the tube again (Lewis et al. 2017; Martin-Ordas et al. 2014; Martin-Ordas et al. 2013). Generally, it seems likely that apes remembered the solution with both tubes (i.e., clear and opaque) because recent studies have shown good long-term memory performance in wild and captive apes, spanning months and even years (Janmaat et al. 2013, 2014, 2016; Kano and Hirata 2015; Lewis et al. 2017; Martin-Ordas et al. 2010, 2013, 2014; Mendes and Call 2014). However, to clearly disentangle these two possibilities one would need to compare latencies, an analysis we were not able to carry out in this study. A recent study performed such an analysis and found that chimpanzees who learned to manufacture an elongated tool three years and seven months before used the same solution strategy and did so faster than during first acquisition and also transferred it to an opaque apparatus (Vale et al. 2016). Future studies will show if this was also the case in the FPT.

One possible explanation for why subjects solved the opaque versions of these tasks after having solved the clear ones is that they were able to recall the effect of their actions despite not seeing it. Alternatively, after solving the task, motor programs alone were capable of sustaining the solution despite the lack of visual feedback. Although this could explain the results, it does not seem enough to explain the results of two simpler tasks employed by Völter and Call (2012) that subjects solved even without the benefit of visual feedback. In these tasks, apes had to either poke out a food reward from a clear or opaque tube or to remove sticks from a clear or opaque tower so that a food reward was released. Although one could argue that the two less complex tasks provide this evidence given that solutions occurred even in the opaque versions that required multiple steps (Völter and Call 2012), the actions required to solve these tasks were relatively simple (insert a stick in a tube or remove sticks from a tower), thus, raising the possibility that subjects may have arrived to them by chance. Furthermore, even those simple actions caused some visible change in the state of the world (e.g., sticks off the box) that the other tasks (crank task, FPT) did not provide.

Some of the apes acquired the solution in the FPT after experiencing a water-filled tube with the peanut floating atop. More specifically, in case of the clear tube two apes benefited from an end-state demonstration and one individual from perceiving how water from a water tap filled the tube until the peanut could be reached (Experiment 2). In case of the opaque tube one subject solved the task and two further individuals added water to the tube after receiving an end-state demonstration once or twice (Experiment 1). Admittedly, this is not a major improvement and we were not able to carry out statistical analyses due to this floor effect, but one has to consider that in case of the clear tube half of our sample comprised previously unsuccessful apes so that chances of them being successful were reduced (Hanus et al. 2011; Tennie et al. 2010). In fact, previous studies have established that subjects who had failed to solve the FPT in the first two sessions were unable to improve if they were simply given additional sessions (Hanus et al. 2011; Tennie et al. 2010). The relative low success attained by subjects even after witnessing the peanut floating upwards suggests that feedback about the water causing the peanut’s movement per se is not a clue that any subject would use to solve the task (contrary to children who would imitate the precise actions required; Nielsen 2013). One possible explanation is that witnessing an effect is less memorable than causing an effect, but when subjects also had the chance to make the tap drop water in the tube this did not increase success rates. Obviously, here the means that they experienced or used themselves (pulling a string to release water from a tap) during the exposure phase and those that they would have to use during the test (pouring water from the mouth) were different, and consequently subjects may have not transferred the solution using different means. Thus, our findings corroborate the ones of Tennie et al. (2010), although we did not explicitly test for imitation versus emulation learning as apes were not given the chance to imitate the precise actions in the first place (i.e., using a bottle to transport water; for a direct comparison see Call et al. 2005; Horner and Whiten 2005; Tennie et al. 2006, 2010; Whiten et al. 2009). Interestingly, Hanus et al. (2011) found that encountering a partial solution (i.e., a quarter-filled tube with the peanut floating atop) did not facilitate the solution for apes as it did for children, perhaps because in the end-state condition apes could access the peanut and touch the water (i.e., were reinforced), whereas they could not do so in the case of the quarter-filled tube. However, future studies are needed to investigate the difference between learning from a partial and a full solution in problem-solving situations more closely.

To sum up, visual feedback can play an important role in great ape problem-solving, especially with complex tasks like the FPT. In the current study, apes required visual feedback for first acquisition of the solution, but could uphold performance thereafter independent of visual feedback. Moreover, some apes acquired the solution after perceiving the end-state, that is, they produced the solution with their own actions. Future research may explore the phylogenetic roots of innovative problem-solving further, involving more diverse tasks and more primate species as well as larger samples.

## Electronic supplementary material

Below is the link to the electronic supplementary material.

Supplementary material 1 (XLSX 45 kb)

Supplementary material 2 (PDF 665 kb)
