# Supplementary material for: Innovative problem solving in great apes: the role of visual feedback in the floating peanut task
Source: Anim Cogn. 2019 Jul 5;22(5):791–805. doi: 10.1007/s10071-019-01275-0 (PMC6687703; doi:10.1007/s10071-019-01275-0)
Supplement: Supplementary file 2 — Supplementary material 2 (PDF 665 kb) [file 10071_2019_1275_MOESM2_ESM.pdf]

Innovative problem solving in great apes: the role of visual feedback in the floating peanut task

Sonja J. Ebel, Martin Schmelz, Esther Herrmann & Josep Call

Online Supplemental Material

Additional tables

Table S1. Subjects participating in Experiment 1.

Name	Species	Sex	Age
Jane	Chimp	Female	11
Joy	Chimp	Female	11
Victoria	Chimp	Female	11
Saidia	Chimp	Female	12
Eva	Chimp	Female	13
Julia	Chimp	Female	15
Mwanzo	Chimp	Female	19
Bahati	Chimp	Female	22
Tess	Chimp	Female	23
Chipie	Chimp	Female	24
Dufatanya	Chimp	Female	24
Amizero	Chimp	Female	26
Akela	Chimp	Female	27
Cheetah	Chimp	Female	28
Roy	Chimp	Male	8
Ali Kaka	Chimp	Male	13
Cumbo	Chimp	Male	15
Edvard	Chimp	Male	15
William	Chimp	Male	15
Zee	Chimp	Male	15
Amihirwe	Chimp	Male	17
Niyonkuru	Chimp	Male	25
Uruhara	Chimp	Male	26
Ndaronse	Chimp	Male	27

Table S2. Subjects participating in Experiment 2.

Subject	Species	Sex	Age	Rearing	Participated in previous studies	Spat water
Fimi	Bonobo	Female	6	Mother	No	NA
Gemena	Bonobo	Female	9	Mother	No	NA
Luiza	Bonobo	Female	9	Mother	No	NA
Lexi	Bonobo	Female	15	Nursery	No	NA
Yasa	Bonobo	Female	17	Mother	No	NA
Kuno	Bonobo	Male	18	Nursery	No	NA
Kara	Chimp	Female	9	Mother	No (with her mother, see Fraukje)	NA[2]
Alexandra	Chimp	Female	15	Nursery	Hanus et al. (2011), Exp. 1 & Exp. 3	No
Annett	Chimp	Female	15	Nursery	Hanus et al. (2011), Exp. 1 & Exp. 3; Tennie et al. (2010)	Maybe[1]
Swela	Chimp	Female	19	Mother	Hanus et al. (2011), Exp. 1 & Exp. 3; Tennie et al. (2010)	Maybe[1]
Sandra	Chimp	Female	21	Mother	Hanus et al. (2011), Exp. 1 & Exp. 3; Tennie et al. (2010)	Maybe[1]
Jahaga	Chimp	Female	21	Mother	Hanus et al. (2011), Exp. 1 & Exp. 3	Yes
Daza	Chimp	Female	28	Unknown	No	NA
Natascha	Chimp	Female	34	Nursery	Hanus et al. (2011), Exp. 1	No
Riet	Chimp	Female	36	Nursery	Hanus et al. (2011), Exp. 1 & Exp. 3	No
Corrie	Chimp	Female	37	Nursery	Hanus et al. (2011), Exp. 1 & Exp. 3	No
Ulla	Chimp	Female	37	Nursery	Hanus et al. (2011), Exp. 1 & Exp. 3	Yes
Fraukje	Chimp	Female	38	Nursery	Hanus et al. (2011), Exp. 1 & Exp. 3; Tennie et al. (2010)	Maybe[1]
Frederike	Chimp	Female	40	Unknown	No	NA
Jeudi	Chimp	Female	48	Unknown	No	NA
Kofi	Chimp	Male	9	Mother	No (with his mother, see Ulla)	NA[2]
Lobo	Chimp	Male	10	Mother	Hanus et al. (2011), Exp. 3	No
Alex	Chimp	Male	13	Nursery	Hanus et al. (2011), Exp. 1 & Exp. 3; Tennie et al. (2010)	Maybe[1]
Robert	Chimp	Male	38	Nursery	Hanus et al. (2011), Exp. 1	No

[1] Maybe in Tennie et al. (2010), not reported.

[2] Kofi has potentially observed how his mother added water to the tube once. Kara has experienced a chimp demonstration, but probably no spitting by her mother. Both were two to three years old at that time.

Table S3. Spitting behaviour in Experiment 2 that did not result in the retrieval of the peanut.

Subject	Species	Age	Session	Conditions	Number of spits
Fimi	Bonobo	6	1, 5	Baseline, water tap by ape	1 (+4)[1], 2
Kara	Chimp	9	2, 3, 4	Baseline, water tap by human (2x)	1, 1, 2
Lobo	Chimp	10	2	Baseline	1
Riet	Chimp	36	1	Baseline	1

[1] Four spits occurred after ten minutes had past (in the end the tube was quarter-filled).

Table S4. Subjects participating in Experiment 3.

Subject	Species	Sex	Age	Rearing	Zoo	Experiment	First condition
Kara	Chimp	Female	9	Mother	Leipzig	2, 3	No visual cause
Tai	Chimp	Female	12	Mother	Leipzig	2, 3	Visual cause
Alexandra	Chimp	Female	15	Nursery	Leipzig	2, 3	Visual cause
Sandra	Chimp	Female	21	Mother	Leipzig	2	-
Kofi	Chimp	Male	9	Mother	Leipzig	2, 3	Visual cause
Lobo	Chimp	Male	10	Mother	Leipzig	2, 3	No visual cause
Lome	Chimp	Male	13	Mother	Leipzig	2	-
Frodo	Chimp	Male	20	Mother	Leipzig	2	-
Tao	Orang	Female	9	Mother	Dortmund	2	-
Raja	Orang	Female	10	Mother	Leipzig	2	-
Padana	Orang	Female	16	Mother	Leipzig	2, 3	Visual cause
Toba	Orang	Female	20	Mother	Dortmund	2, 3	No visual cause
Dokana	Orang	Female	25	Mother	Leipzig	2	-

Additional figure

Fig. S1 The activated water tap in the water tap by ape condition (A) and the water tap by human condition (B) is shown from outside the test room in Experiment 2

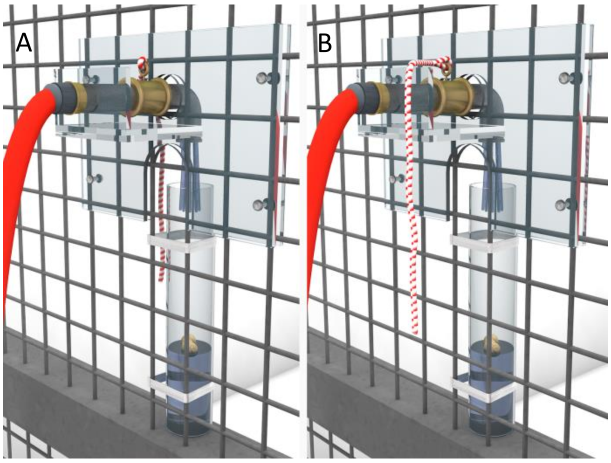

Additional information on Experiment 3

Long-term memory

Subjects acquired the solution in the FPT at different times. Toba was tested nine years ago while her six-month-old daughter Tao was present (Mendes, Hanus, & Call, 2007). Padana and Dokana were also tested nine years ago (Mendes et al., 2007) and re-tested five years age and two years ago respectively. Dokana additionally participated in several TV documentaries, but did so for the last time about two years prior to the current study. Two-year-old Raja was present when her mother Pini was tested in the original study nine years ago (Mendes et al., 2007). Subsequently, Pini was re-tested two years ago and Raja was tested for the first time two years ago as well. Pini was not tested in the current study because she could not be separated from her current offspring whom we wanted to give the task later as well. Frodo was trained to function as a demonstrator about seven years ago (Tennie, Call, & Tomasello, 2010). Lome and Tai were also tested about seven years ago (Hanus, Mendes, Tennie, & Call, 2011). Tai additionally participated at least in one TV documentary one and a half years ago. Alexandra, Kara, Kofi, Lobo and Sandra were tested one month prior to Experiment 3 (mean: 29 days, minimum: 24 days, maximum: 33 days), but had only solved the task once, whereas the others had solved it multiple times.

As Raja, Dokana and Padana had already been re-tested, they were not given a reminder with the clear tube, but started with the opaque tube straight away. For reasons of consistency we

should have treated the chimpanzee Tai in the same way because she had been re-tested for a TV documentary, but we were not aware of this fact at the time of conducting the study.

Comment on the testing of Toba, Tao and Padana

Toba and Tao had been transferred to a different holding facility since the last study was conducted (Mendes et al., 2007). Unfortunately, the clear tube did not fit well to the mesh of the sleeping room at the new facility so that a rectangular plate at the bottom of the tube protruded. Toba bit it off in her first session accessing the peanut in this way. We repeated the session on the next day with a tube that was closed at the bottom but lacking a rectangular plate. The caregivers reported beforehand that the orang-utans should probably know about the water dispensers in their sleeping rooms, although they had rarely seen them drinking from these dispensers. Especially with Tao they were unsure if she was actually aware of this drinking option. Thus, in her first session, we provided Tao with a bowl of water which she spilled after a while so that we do not know if she had a chance to solve the task at all. Before Toba broke off the bottom of the tube in her first session, she spat saliva into the tube several times so that we were also not sure if she was aware of the water dispenser. To give them a fair chance, we provided both with a large bucket of water in the second session that we placed on the floor of the test room and then, both orang-utans solved the task.

For Toba and Tao, the mesh surrounding the opaque tube could not be covered completely due to the conditions at the holding facility. However, we covered the hose at the back of the tube that led to a basin to collect the water. Importantly, the setup looked exactly the same for the opaque condition, the “visual cause” and the “no visual cause” condition from the perspective of the ape. However, the hose was not yet cut on the surface to let the air out so that Toba experienced a gurgling sound in the no visual cause condition when the water flew out. Padana was tested with the same setup as Toba in the first two sessions of the opaque condition. She did not solve the task and after receiving reminder sessions with the clear tube, she received the opaque tube again, but this time the mesh surrounding the tube was completely covered as it was for all the other apes.

References

Hanus, D., Mendes, N., Tennie, C., & Call, J. (2011). Comparing the performances of apes (*Gorilla gorilla, Pan troglodytes, Pongo pygmaeus*) and human children (*Homo sapiens*) in the floating peanut task. *PLoS One, 6*(6), e19555. doi:10.1371/journal.pone.0019555

Mendes, N., Hanus, D., & Call, J. (2007). Raising the level: orangutans use water as a tool. *Biol Lett, 3*(5), 453-455. doi:10.1098/rsbl.2007.0198

Tennie, C., Call, J., & Tomasello, M. (2010). Evidence for emulation in chimpanzees in social settings using the floating peanut task. *PLoS One, 5*(5), e10544. doi:10.1371/journal.pone.0010544
